# Recombinant scFv-Fc Anti-kallikrein 7 Antibody-Loaded Thermosensitive Hydrogels Against Skin Desquamation Disorders

**DOI:** 10.1021/acsabm.4c00371

**Published:** 2024-06-18

**Authors:** Ana Flávia Santarine Laureano, Aryane Alves Vigato, Luciano Puzer, Daniele Ribeiro de Araujo

**Affiliations:** † Department of Surgery, Center for Transplantation Sciences, Massachusetts General Hospital & Harvard Medical School, CNY149 13th Street, Charlestown, Boston, Massachusetts 02129, United States; ‡ Centro de Ciências Naturais e Humanas, Universidade Federal do ABC, Al. da Universidade, s/n-Anchieta, São Bernardo do Campo, SP 09606-045, Brazil; § Department of Biomedical Science (BMV), Faculty of Health and Society, Malmö University, Malmö 20506, Sweden; ∥ Biofilms−Research Center for Biointerfaces, Malmö University, Malmö 20506, Sweden; ⊥ Centro de Ciências Naturais e Humanas, Universidade Federal do ABC, Av. dos Estados, 5001, Bloco A, Torre 3, Santo André, SP 09210-580, Brazil; # Departamento de Biofísica, Escola Paulista de Medicina, Universidade Federal de São Paulo, Rua Botucatu, 862, Vila Clementino, Sao Paulo, SP 04023-062, Brazil

**Keywords:** Pluronic, scFv-Fc antibodies, kallikreins, atopic dermatitis, hydrogel

## Abstract

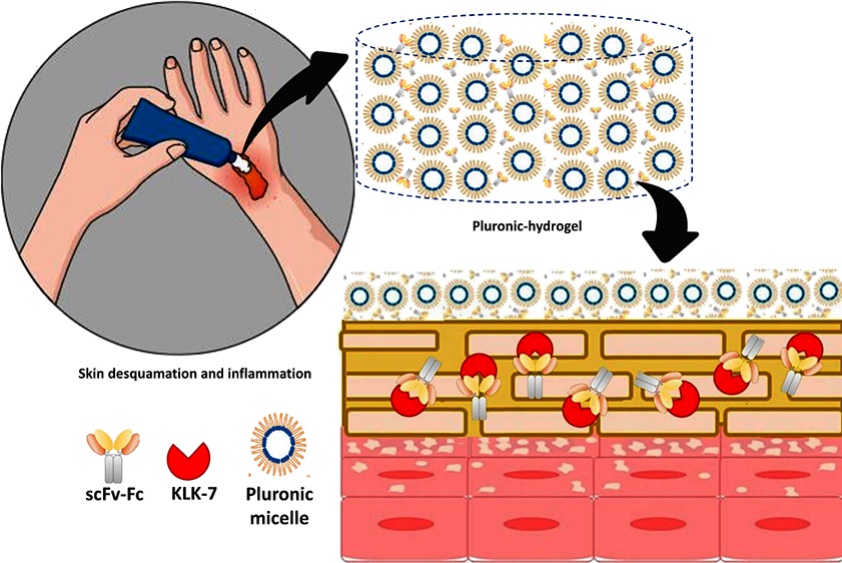

Human tissue kallikrein-related peptidase 7 (KLK7) is a serine protease implicated in the physiology of skin desquamation, and its uncontrolled activity can lead to chronic diseases such as psoriasis, atopic dermatitis, and Netherton syndrome. For this reason, kallikrein 7 has been identified as a potential therapeutic target. This work aimed to evaluate Pluronic (PL) hydrogels as topical carriers of four specific scFv-Fc antibodies to inhibit KLK7. The hydrogels comprised PL F127 (30% w/v) alone and a binary F127/P123 (28–2% w/v) system. Each formulation was loaded with 1 μg/mL of each antibody and characterized by physicochemical and pharmaceutical techniques, considering antibody–micelle interactions and hydrogel behavior as smart delivery systems. Results showed that the antibodies were successfully loaded into the PL-based systems, and the sol–gel transition temperature was shifted to high values after the P123 addition. The antibodies released from the gels preserved their rheological properties (*G*′ > *G*′′, 35- to 41-fold) and inhibitory activity against KLK7, even after 24 h. This work presented potential agents targeting KLK7 that may provide strategies for treating skin abnormalities.

## 1 Introduction

Monoclonal antibodies (mAbs) have been used to treat various diseases and are emerging as a significant class of protein therapeutics on the market. Recently, the FDA approved 12 new mAbs, constituting 20% of the agency’s approved drugs. Out of these, at least half are expected to achieve blockbuster drug status, yielding annual sales of US$ 1 billion or more by 2024.^1^ Since the advent of hybridoma technology, researchers and the pharmaceutical industry have quickly recognized the potential of antibodies as therapeutic agents. Over the years, the technologies for producing antibodies have evolved in order to reduce immunogenicity usually by means of chimerization or humanization of mouse monoclonal antibodies.^2^

Among the methods for human antibody generation, the transgenic mice^3^ and *in vitro* display technologies can be highlighted.^4^ Antibody phage display is the most employed method for *in vitro* selection due to its robustness and easy-to-perform and well-developed experimental methodologies of bacteriophage use.^2^ Libraries used for antibody phage display are sources of fully human antibodies, antibody fragments (i.e., Fab or scFv), and antibodies from natural or synthetic origin.^5^ These platforms represent a catalog of drug/therapeutic molecules and target-neutralizing agents.^2^ In this context, phage display technology contributes to the release of many commercialized antibody drugs.^5,6^

Skin desquamation is a natural process mediated by the action of enzymes known as human tissue kallikrein-related peptidases (KLKs).^7−12^ Among these proteases, KLKs 5 and 7 are highly expressed in the epidermis by granular keratinocytes and are present in the intercellular matrix of the stratum corneum (SC).^10^ In fact, there is an intricate regulatory mechanism of skin desquamation involving KLKs 5 and 7. KLK 7 is activated by KLK 5, and its *in vivo* activity is, additionally, regulated by specific inhibitors, such as the lympho-epithelial Kazal-type-related inhibitor (LEKTI).^13^

The unregulated actions of both KLKs 5 and 7 result in Netherton syndrome (NS) development, a rare autosomal recessive disease that triggers severe skin inflammation and scaling, since the loss of the inhibitor LEKTI activity is responsible for epidermal protease regulation.^14^ Another important point is that the mutations in SPINK5 (serine protease inhibitor of Kazal-type 5) encoding LEKTI cause hair shaft defects and constant allergic manifestations. Additionally, NS is also associated with many dermatological disorders such as psoriasis, atopic dermatitis,^15^ acne rosacea,^16^ and even melanoma,^17^ which are caused by aberrant expression and/or a lack of inhibition of KLKs in the SC. All those factors are linked to defects in the ability of the skin to maintain its homeostasis and so far have no cure.^7,18^

In this sense, there is a crucial role involving KLKs 5 and 7, the inhibitor LEKTI, and its SPINK5 encoding gene, which motivated investigations *in silico* and *in vivo*. For example, Furio et al.^19^ developed a KLK5/SPINK5 knockout murine model reporting that the KLK5 deletion resulted in Th17 response blockade. Similar strategies also highlighted the *in vivo* pathways related to the skin protease roles in NS.^14^

Many groups are currently investigating different strategies for treating these skin conditions, especially NS and atopic dermatitis. Jendrny and Beck-Sickinger have found compounds based on a sunflower trypsin inhibitor (SFTI) with an inhibitory effect over KLKs 5 and 7.^20^ The SFTI strategy was previously described by de Veer et al.^21^ when they performed a structure–activity analysis for selecting adequate SFTI homologues capable of recognizing different surfaces in the protease active site. In another report, the same research group expanded the study for KLK5, KLK7, and KLK14 inhibitors, resulting in a detailed description of the KLK role in the cutaneous barrier.^22^ More recently, Chavarria-Smith et al.^23^ developed human antibodies with high *in vivo* capacity for simultaneously inhibiting both proteases, KLK5 and KLK7, providing an innovative strategy for the treatment of inflammatory dermatoses.

Our research group has published reports of inhibitors for both KLKs 5 and 7 based on natural products,^24,25^ synthetic compounds,^26−28^ and proteins.^29^ In addition, a generation of scFv antibodies, using phage display technology with inhibitory power over KLK7, was recently published.^30^ To the best of our knowledge, this was the first time that phage display technology has been used for producing antibody fragments (scFv) against KLKs, showing a short range of molecular inhibition (6.2–0.9 nM).

Biologicals (e.g., proteins, peptides, and antibodies) have shown great importance in medicine to manage or reverse the course of diseases. However, guaranteeing the stability of these molecules in pharmaceutical formulations is still a challenge.^31,32^ In this way, drug delivery systems have been developed to decrease the degradation of bioactive molecules, avoid side effects, and enhance the drug amount in the target. There are several materials and chemical strategies used to develop drug delivery systems such as liposomes, nanoparticles, and micelles.^33^ Pluronics (PLs) have been demonstrated as promising polymers to compose drug delivery systems due to their thermosensitive properties, high biocompatibility, and variety in the market.^33^ PLs are a class of water-soluble nonionic triblock polymers, composed of a central block of poly(propylene oxide) (PPO) and two side blocks of poly(ethylene oxide) (PEO). The PL monomers can self-organize into micelles above critical concentrations, and these micelles can further interact, forming gels in response to the temperature.^34,35^ Moreover, PL-based hydrogels showed suitable viscoelastic properties for topical applications, which ensures even distribution of the product,^36,37^ and have been demonstrated to improve the permeation of small molecules through the skin.^38−40^

In this context, this work focuses on the preparation of PL-based hydrogels using PL F127 and P123 to encapsulate the scFv-Fc antibody anti-KLK7 (previously characterized by Laureano et al.^30^) and on the evaluation of the permeation profiles of these biologicals. The inhibitory effect toward KLK7 of the antibodies was assessed to verify if they remained active after the permeation. Since KLKs are important therapeutical targets, this work aims to develop new formulations for treating topical diseases involving these proteases’ activity.

## 2 Materials and Methods

### 2.1 Chemicals

Pluronic F127 (poloxamer 407) and Pluronic P123 (poloxamer 403) were purchased from Sigma-Aldrich (MO, USA). All other chemicals and solvents were of analytical grade. The generation of the four scFv-Fc antibodies LUP-37A10 (A10), LUP-37B10 (B10), LUP-37C11 (C11), and LUP-37D11 (D11) studied in this work was described by Laureano and co-workers.^30^ The production of recombinant KLK7 was described by De Souza et al.^41^ The antibody sequences and structural details are provided in the Supporting Information.

### 2.2 Antibody-Loaded Pluronic Hydrogel Preparation

For this study, two compositions of Pluronic hydrogels were prepared: PL F127 30 % w/v (F127) and a binary system containing PL F127 28 % w/v and PL P123 2 % w/v (F127/P123). First, polymers were homogenized in ultrapure water in an ice bath until complete dissolution was achieved (cold method) and were stored at 8 °C for 24 h.^42^ The antibodies were dispersed into the solutions and kept at 4 °C with magnetic stirring (100 rpm). After complete dissolution, each formulation was equilibrated overnight at 4 °C. The final concentration of the antibodies in the hydrogels was 1 μg/mL.

### 2.3 Micellar Hydrodynamic Diameter and Average Size Distribution

The dynamic light scattering (Zetasizer Nano ZS, Malvern Panalytical Ltd., UK) technique was used to characterize the micellar hydrodynamic diameter and the average size distribution. Data were obtained at a fixed angle of 173° and temperatures settled at 25 and 32.5 °C (skin temperature). Polystyrene cuvettes (10 nm, 4.5 mL) were filled with 1 mL of each micellar solution (5%, with and without antibodies). Hydrodynamic micellar diameters were measured at least five times for each sample.

### 2.4 Differential Scanning Calorimetry

The micellization temperature (*T*_m_) and enthalpy variation (Δ*H*°) referred to PL micellization were obtained from calorimetric analysis (Q-200 calorimeter, TA Instruments, USA). Adequate amounts of hydrogels (F127 and F127/P123) were added into hermetic aluminum pans and submitted to thermal cycles of heating–cooling–heating from 0 to 50 °C at a 5 °C/min rate. The resulted thermograms were represented by heat flow (W/g) versus temperature (°C).

### 2.5 Rheological Analysis

To determine the sol–gel temperature transition (*T*_sol–gel_), rheological analysis was performed by using an oscillatory rheometer (Kinexus Lab, Malvern Panalytical Ltd., UK) with a cone–plate geometry (20 mm diameter, 0.5 rad angle, and 1 mm gap). The apparent viscosity (η*) and elastic (*G*′) and viscous moduli (*G*″) were the rheological parameters evaluated. For all analysis, 1 mL of each formulation was added to the sample holder, and two methods were performed: (i) a temperature sweep from 10 to 50 °C at 5 °C/min and (ii) oscillatory analyses under frequency sweep from 0.1 to 10 Hz at 32.5 °C. All data were analyzed by using rSpace for Kinexus and GraphPad Prism software (Prism 5.0, GraphPad Software, Inc., USA).

### 2.6 *In Vitro* Release Assays

*In vitro* assays were performed to evaluate the antibodies’ release profiles from the hydrogels by using a membrane-less model, allowing the contact of the formulation with the receptor medium.^43^ A donor cell (0.8 and 2.5 cm of diameter and height, respectively) was filled with hydrogel samples (1 g) and placed in a glass receptor compartment (50 mL) containing a 5 mM phosphate buffer, at pH 7.4. A magnetic stirring bar was placed into the receptor compartment, and the system was maintained under constant stirring (350 rpm) at 32.5 °C for 24 h. At regular intervals (0.5, 1, 2, 4, 6, 8, and 24 h), 1 mL of the receptor medium was withdrawn, and the antibody content was quantified by ELISA.

### 2.7 Assessing the scFv-Fc Inhibitory Effect over KLK7

For assessing the antibodies’ inhibitory effects over KLK7, the enzyme activity was monitored by hydrolysis of a fluorogenic substrate in a Biotek Synergy H1 microplate reader (Agilent Technologies, USA). Excitation and emission wavelengths were adjusted to λ_ex_ = 380 nm and λ_em_ = 460 nm, respectively. The assays were performed in a black microtiter plate (Greiner Bio-One, Frickenhausen, Germany) with a final volume of 200 μL. Buffer solutions (Tris 50 mM, pH = 7.5) with the enzyme and inhibitor were kept at 37 °C for 2 min before the addition of the fluorogenic substrate Abz-KLYSQ-EDDnp (GenOne, Brazil) at a 10 μg/mL final concentration. Positive controls (KLK7 enzyme in the absence of its inhibitors) were evaluated by monitoring the activity of KLK7 by releasing fluorescence as a function of time for 10 min. The slope was converted into nanomoles of the hydrolyzed substrate per minute, based on the fluorescence curve of the peptide solution with a known concentration. scFv-Fc antibodies were used as inhibitors and were added to the assay at known concentrations after 24 h of release.

### 2.8 *In Vitro* and *Ex Vivo* Permeation Assays

Permeability experiments across the epithelial barrier were performed by using an automated vertical diffusion system (Microette Plus, Hanson Res., City of Industry, CA, USA) with a permeation area of 1.72 cm^2^. Artificial membranes (Strat-M, Merck Millipore, Germany) and porcine ear epidermis were used as *in vitro* and *ex vivo* skin models, respectively. All protocols were approved by The Animal Ethical Committee of Federal University of ABC (no. 1130300119). Franz cell receptor compartments were filled with 7 mL of a 5 mM phosphate buffer, at pH 7.4. The systems were maintained at 32.5 °C under constant stirring (350 rpm).^44^ Samples were collected from the receptor compartment at 0.5, 1, 2, 4, 6, 8, and 24 h, and antibody concentrations were determined by ELISA (*n* = 6/formulation).

## 3 Results and Discussion

### 3.1 Micellar Characterization: Hydrodynamic Size and Micellization Temperature

**Figure 1:**
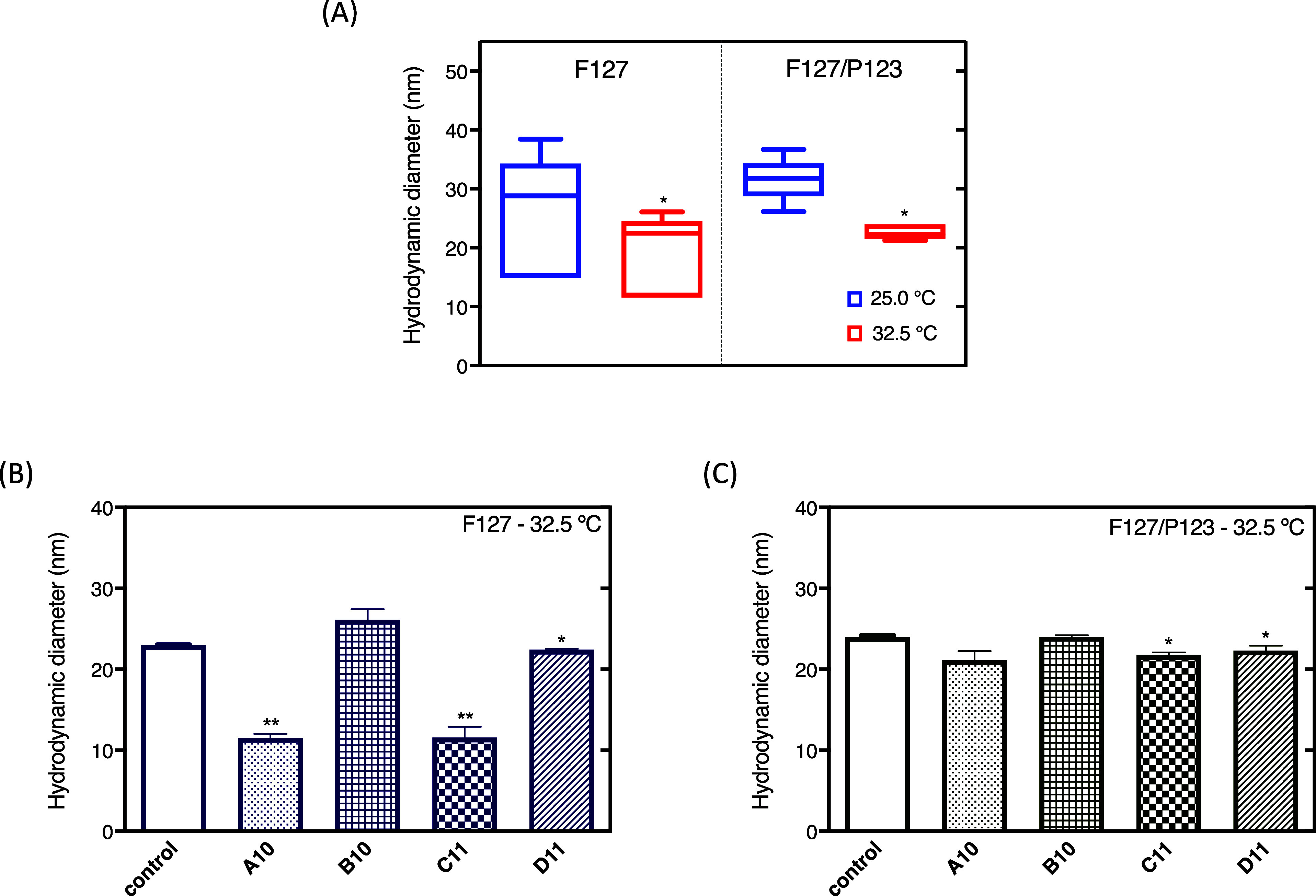
Influence of the temperature on the distribution of micellar hydrodynamic diameter average values, including all hydrogels (F127 and F127/P123 with and without antibodies) (A). Effect of the antibody addition into the F127 (B) and F127/P123 (C) systems. 25 **°**C vs 32.5 **°**C (*p* < 0.05) [*] by *t*-tests (A). Control vs micelles with antibodies *p* < 0.01 [**] and *p* < 0.05 [*] by ANOVA (one-way)–Dunnett tests (B,C). Measurements obtained for F127 and F127/P123 systems at 5% (w/v).

The micellar hydrodynamic diameter and the average size distribution were evaluated by dynamic light scattering (DLS) in the presence and absence of scFv-Fc antibodies (Table 1). In general, the hydrodynamic diameter variation showed a temperature dependence, in which the increase in temperature leads to a decrease in the micellar diameter (Figure 1A). For instance, the F127 micellar hydrodynamic diameters were 25.4 ± 10.9 and 18.9 ± 6.1 nm at 25 and 32.5 °C, respectively, while for the F127/P123 system, values were from 31.6 ± 3.34 nm at 25 °C to 24.5 ± 4.6 nm at 32.5 °C. Regarding the distribution profiles, a small population (3 to 5%) of particles with diameters of <5 nm was found at 25 °C, probably corresponding to unimers that were not identified at 32.5 °C, as expected.^45^ Moreover, the hydrodynamic diameter variation can be explained by the promotion of the propylene oxide (PPO) unit dehydration in the micellar core in response to a temperature increase.^46^ This temperature effect also influenced the hydrophobicity of the micellar core, favoring the formation of colloidal systems with low polydispersity (PDI = 0.15 to 0.31). Similar results were also reported for F127 micelles and their association with more hydrophobic PL.^47−50^

**Table 1:** Hydrodynamic Diameter (nm) and Polydispersity Index (PDI) of F127 and F127/P123 Hydrogels in the Presence and in the Absence of the Antibodies A10, B10, C11, and D11 at Temperatures of 25 and 32.5 °C (*n* = 5)

	**25 °C**		**32.5 °C**	
**Hydrogel**	**Hydrodynamic diameter (nm)**	**PDI**	**Hydrodynamic diameter (nm)**	**PDI**
F127	28.8 ± 0.9	0.46 ± 0.05	23.0 ± 0.1	0.31 ± 0.01
F127/P123	31.8 ± 0.3	0.40 ± 0.05	24.0 ± 0.2	0.26 ± 0.01
F127-A10	14.9 ± 0.3	0.26 ± 0.20	11.6 ± 0.5	0.15 ± 0.10
F127/P123-A10	31.3 ± 1.1	0.30 ± 0.01	21.1 ± 1.1	0.16 ± 0.03
F127-B10	38.4 ± 7.3	0,43 ± 0,10	26.1 ± 1.3	0.31 ± 0.01
F127/P123-B10	36.7 ± 2.5	0.30 ± 0.05	24.0 ± 0.2	0.29 ± 0.02
F127-C11	14.9 ± 1.3	0.46 ± 0.05	11.6 ± 1.3	0.31 ± 0.01
F127/P123-C11	32.0 ± 0.3	0.34 ± 0.01	21.8 ± 0.3	0.23 ± 0.01
F127-D11	30.2 ± 0.1	0.39 ± 0.05	22.5 ± 0.1	0.29 ± 0.01
F127/P123-D11	26.1 ± 0.7	0.28 ± 0.01	22.3 ± 0.6	0.16 ± 0.10

Previous studies have reported that the combination of PL F127 and P123 can improve micellar properties as drug delivery systems, such as increased stability and biocompatibility, compared with isolated PL 123 micelles.^51,52^ Ganguly et al. reported a structural study by small-angle neutron scattering (SANS), showing that F127 and P123 could form mixed micelles due to the similar PPO block length (∼62 PPO units for both PLs), and their core radius decreases with the incorporation of F127.^51^ However, our results did not show a significant difference between the F127 micellar size and the binary system F127/P123 at the studied temperatures. These differences can be explained by the small amount of P123 added to F127, which could not promote expressive changes in micellar dimensions. In this context, the study of the diluted formulations by DLS can contribute to understanding antibody–micelle interactions.^47,53^ At 32.5 °C, the incorporation of A10, C11, and D11 antibodies significantly decreased the hydrodynamic diameter of the F127 micelles (Figure 1B). C11 and D11 also promoted the same effect in the binary systems (Figure 1C). In general, the effects of the antibody incorporation were more pronounced for F127 systems than for F127/P123. Those possible micellar dimension changes can be due to an equilibrium shift of F127 molecules (component with the highest concentration in the systems). In fact, the distribution profiles of micellar dimensions are (in part) composed of a small population (3 to 5%) of particles with diameters of ∼5 nm, probably corresponding to polymer unimers. Then, F127 unimers can be shifted for interacting with the antibodies (instead of self-assembled in micelles), which does not exclude the possibility of mixed micelle coexistence and even different phase organizations (considering hydrogels), as previously described by our group in the literature, after hydrogel analysis by SANS.^53^

From calorimetric analysis, the micellization temperature (*T*_m_) and enthalpy (Δ*H*) were determined for both systems, PL F127 and PL F127/P123. The endothermic peak was centered at 12.41 °C for F127, while for F127/P123, it was 14.85 °C. Moreover, the Δ*H* values were 4.986 and 1.524 J/g for F127 and F127/P123, respectively. The differences on the micellization peak between F127/P123 and F127 systems mean that less energy was required for the binary system micellization process, which can be attributed to the possibility of binary micelle formation guided by the fact that both PLs share similar PPO block length and the concomitant formation of unique P123 and F127 micelles during the micellization process. These scenarios involve creating new chemical interactions between the PLs that can affect the *T*_m_ compared to isolated F127, for instance, steric and concentration effects, since F127 is present in 28% of the binary systems.

### 3.2 Rheological Studies

**Figure 2:**
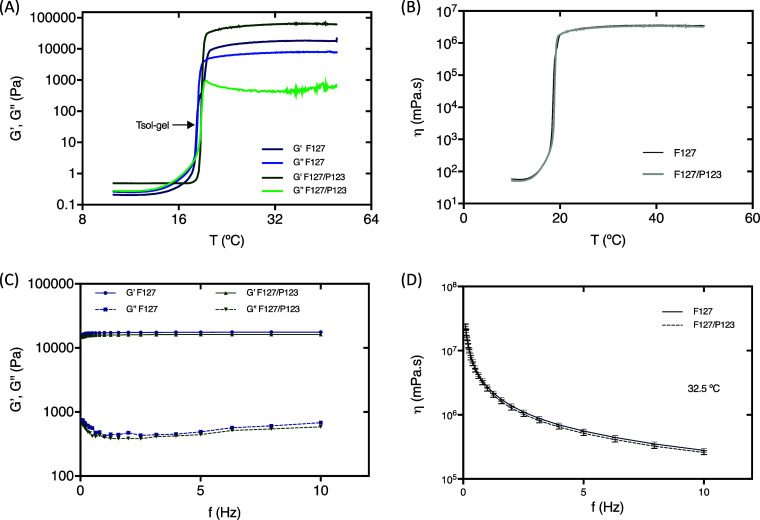
Temperature-dependent (A,B) and frequency-dependent (C,D) rheological analysis of PL F127 and F127/P123 hydrogels without antibodies. Storage (*G*′) and loss (*G*″) modulus (A) and viscosity (B) as a function of temperature. The formulations were heated from 10 to 60 °C at 5 °C min^–1^ under 1 Hz, and *T*_sol–gel_ was defined as the intersection of storage *G*′ and loss modulus *G*″ (indicated by the arrow). Frequency sweep 0.1 → 10 Hz vs *G*′ and *G*′′ (C) and viscosity (D) at 32.5 °C. All formulations presented similar patterns and maintained their integrity without phase separation.

Rheological analyses were performed to evaluate the viscoelastic behavior of the hydrogels. The storage/elastic and loss/viscous moduli (respectively *G*′ and *G*″) and the viscosity (η*) were determined to characterize the sol–gel temperature and the frequency dependence behavior. Both parameters are essential for studying the hydrogels' thermoresponsive properties since during the sol–gel transition, *G*′ and η* undergo a critical variation that characterizes the gelation process. Additionally, the frequency sweep (0.1 → 10 Hz) was used to evaluate the contributions of elastic (*G*′) and viscous (*G*′′) moduli to the frequency dependence of the hydrogels and their capability to preserve their mechanical properties after deformation. The magnitude of *G*′ and *G*′′ values indicates how pronounced the elastic or viscous characteristics are in a gel during the shear.^54^ In this context, Table 2 shows the rheological parameters for all formulations, and Figure 2 shows representative rheograms of formulations F127 and F127/P123 without antibodies.

**Table 2:** Rheological Characterization of the Formulations Based on Antibody A10-, B10-, C11-, and D11-Loaded F127 and F127/P123 Hydrogels^a^

		**32.5 °C and1 Hz**			
**Formulation**	***T*_sol–gel_ (°C)**	***G*′(mPa)**	***G*′′(mPa)**	***G*′/*G*′′**	η*(10^**6**^ **mPa s)**
F127	17.94 ± 0.50	17.960	479.4	37.4	2.525
F127/P123	19.20 ± 0.90	16.360	421.7	38.8	2.658
F127-A10	16.90 ± 2.30	17.420	480.0	36.3	2.569
F127/P123-A10	19.26 ± 1.29	18.700	523.3	35.8	2.492
F127-B10	19.57 ± 1.42	18.170	517.5	35.1	2.894
F127/P123-B10	19.98 ± 1.03	18.600	449.5	41.4	2.961
F127-C11	18.90 ± 0.65	18.240	610.9	29.9	2.904
F127/P123-C11	19.01 ± 1.55	16.520	411.3	40.2	2.630
F127-D11	19.34 ± 1.16	15.260	424.1	36.0	2.430
F127/P123-D11	20.46 ± 1.03	12.840	308.5	41.6	2.044

a Note: *G*′ is the storage modulus, *G*′′ is the loss modulus, and η* is the apparent viscosity.

In general, binary systems showed higher *T*_sol–gel_ values (19.38 ± 0.62 °C) compared with the F127 formulations (18.53 ± 1.10 °C). However, the effect of P123 addition was discrete regarding the *T*_sol–gel_ (Figure 2A) and viscosity (Figure 2B). Similar results were obtained for the formulations containing antibodies, which did not promote significant changes in the *T*_sol–gel_ and the viscosity. Additionally, the *G*′ values were not affected in magnitude by the addition of the antibodies. In this way, the hydrogels containing P123 and the antibodies presented a gel structure similar to the control (F127).

All hydrogels showed a viscoelastic behavior, with *G*′ predominating over *G*″ in which the addition of P123 increased the contribution of *G*′, more than 40 times for the formulations containing B10, C11, and D11. This observation can be attributed to their physicochemical properties, such as a lower HBL value (HBL = 8) compared to F127 (HBL = 22) that can increase the hydrophobic interactions among hydrogel components. In general, *G*′ > *G*′′ indicates that the hydrogel structure is preserved even when the frequency increases; that is, the intermolecular interactions among the micelles are sufficiently strong to hold them together and resist high-shear stress conditions. Moreover, all formulations showed low *G*′ and *G*′′ dependency over the frequency range, indicating hydrogel stability (Figure 2C).

Additionally, in Figure 2D, the viscosity decreases as the function of the frequency can be verified. The literature has reported that PL F127 shows a pseudoplastic behavior in concentrated solutions (>20% w/v), in which the viscosity decreases with the increase in the shear rate.^55^ Our results corroborate this observation. It is worth noting that pseudoplasticity is highly desirable in topical formulations, as it guarantees even product distribution on the skin. This property means that the viscosity of the gel decreases during spreading, ensuring a smooth and consistent skin coverage.^56^

The skin range temperature, usually from 32 to 35 °C, can be affected according to the body region.^57^ This way, considering that the hydrogels may be applied directly to the skin, the antibodies would already be encapsulated into the gel, and the formulations undergo the application stress. The rheological characteristics found here are advantageous for the intended use due to *T*_sol–gel_ below the skin temperature, the high storage modulus (*G*′) contribution, and the hydrogel pseudoplasticity. These properties guarantee a gel with sufficient strength to prevent phase separation and facilitate the application.^53^

### 3.3 *In Vitro* Release Studies and Inhibitory Effect of the scFv-Fcs

**Figure 3:**
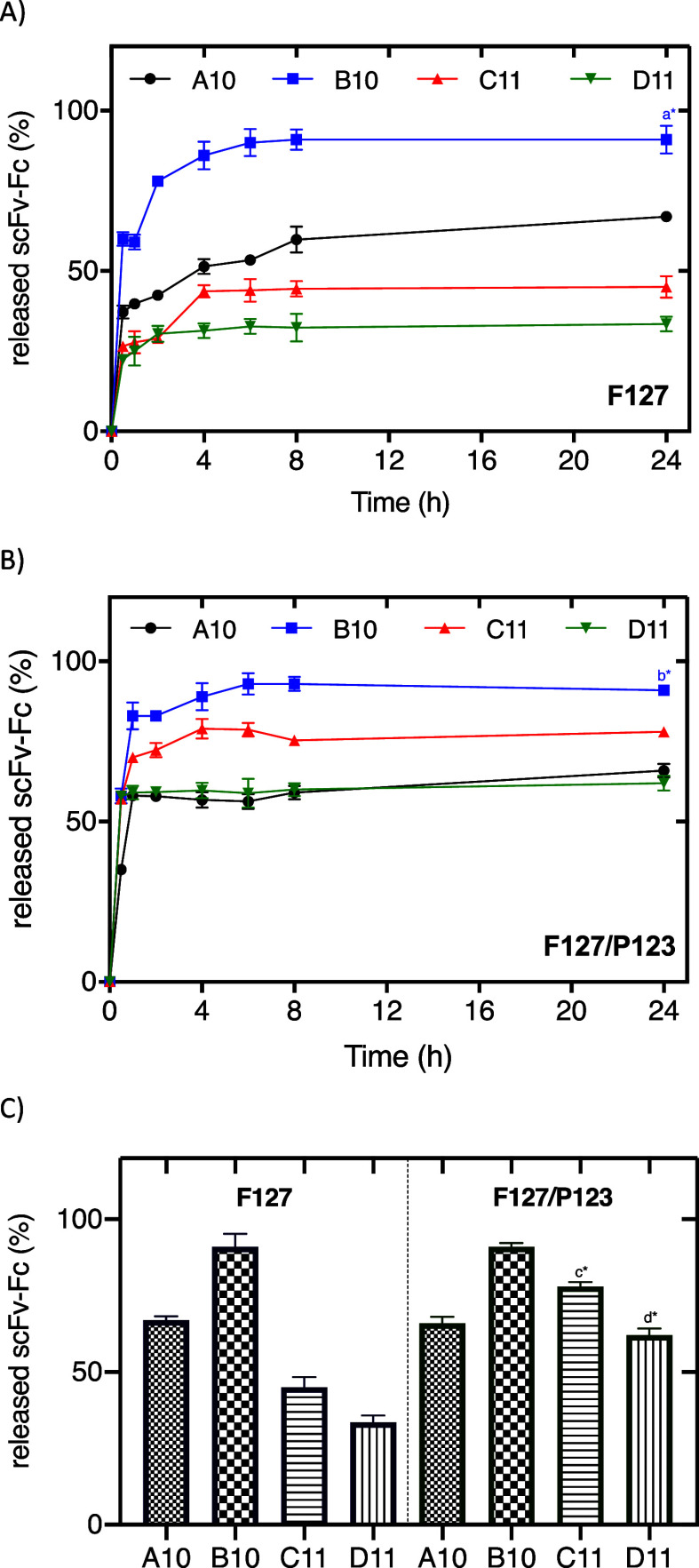
Release profile of antibodies (A10, B10, C11, and D11) from the hydrogels composed of Pluronic F127 30% w/v (A) and F127/P123 28–2% w/v (B) for 24 h. Comparison graph between the antibodies released (at 24 h) from hydrogels composed by F127 and the binary systems F127/P123 (C). Quantification was performed by a modified in-house indirect ELISA assay. B10 vs A10, C11, and D11 [a,b*], C11-F127 vs C11-F127/P123 [c*], and D11-F127 vs D11-F127/P123 [d*] (*p* < 0.0001 [*]) by ANOVA (one-way)–Tukey’s multiple comparison tests.

*In vitro* assays were used to investigate the antibody release profiles from the hydrogels. Figure 3 displays the permeation profiles of all hydrogels composed of F127 (Figure 3A) and F127/P123 (Figure 3B). For both formulations, F127 and F127/P123, antibody B10 showed the fastest release up to 8 h, reaching ∼91% of antibody release for F127 and F127/P123, after 24 h. On the other hand, the antibody D11 showed the slowest release, reaching 33.5% for the F127 hydrogel and 62% for the binary system at the end of the analysis.

All release profiles were analyzed by mathematical models (Higuchi, Korsmeyer–Peppas, and Hixson–Crowell) to calculate the release constants (*K*_rel_) from hydrogels and investigate their mechanism. Equations and mathematical models are detailed in the Supporting Information.

In general, *K*_rel_ constant values for F127-based formulations followed the Korsmeyer–Peppas model and were similar, ranging from 0.1 to 0.17% h^–*n*^, with *n* values of ∼1.4. On the other hand, for the binary composition, F127–P123, the best fitting was obtained by the Higuchi model (0.82 > *R*^2^ > 0.98) with the lowest *K*_rel_ for D11 (3.8% h^–1/2^) release from the hydrogel formulation. From these results, it is possible to postulate that scFv-Fc release mechanisms from F127 are based on relaxation of polymeric chains and further water molecule diffusion, while the F127–P123 system is controlled by diffusion. Those differential mechanisms can be explained by hydrogel composition since the incorporation of P123 (a more hydrophobic polymer compared to F127) possibly reduced the system water solubility and partially maintained its gel structure integrity.

The influence of the P123 presence on the antibody release profiles can be verified in Figure 3C. Additionally, the C11 and D11 released amounts were increased by adding P123 into the hydrogels. These results indicate the hydrophilic character of these antibodies, in which the increase of the gel’s hydrophobicity by adding P123 contributed to disturbance of the affinity of these molecules and their dispersion into the gel matrix.

Materials loaded with scFv-Fc are most commonly applied for cancer therapies.^58,59^ Few reports in the literature use Pluronic-based formulations to carry scFv antibodies. Choi and collaborators prepared nanogels composed of PL F127 conjugated with heparin and used them as carriers of scFv antibodies.^60^ However, the release profile was investigated by dialysis, which means that the gels were diluted, and therefore, a direct comparison with our results would not be accurate. Another study investigated hydrogels containing 19% F127 to carry monoclonal antibodies. Using vertical Franz diffusion cells and a synthetic cellulose acetate membrane, the authors reported that 65% of the antibodies were released from the gel to the receptor media, and the PL hydrogel promoted the release rate control in comparison to other formulation types and compositions, such as creams.^61^

**Figure 4:**
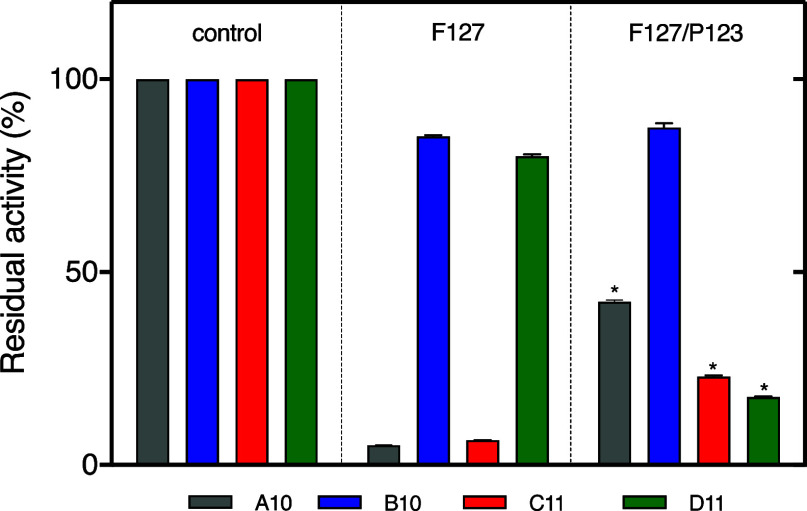
A10, B10, C11, and D11 inhibitory residual activity at 24 h (B) against KLK7. F127 vs F127/P123 (*p* < 0.0001 by ANOVA (two-way)–Tukey’s multiple comparison tests (*n* = 5).

The scFv-Fc potential inhibitory effect against KLK7 was simultaneously evaluated with the release content. KLK7 without any inhibition was set as the positive control for 100% activity, and the scFv-Fcs released from the hydrogels (after 24 h) were added to the assay. The IC_50_ values for the antibodies, previously assessed by our research group, were 0.90 (A10), 6.2 (B10), 0.5 (C11), and 0.8 nM (D11).^30^ The residual activity results are listed in Figure 4. For F127 hydrogels, A10 and C11 exhibited the highest inhibition activity (corresponding to concentrations ranging from 0.7 to 1.2 nM), while for hydrogels composed of F127/P123, C11 and D11 were the best inhibitors (released concentrations from 1.1 to 1.3 nM). Although the available amount of B10 reached almost 100% in the release assays (∼1.65 nM), this antibody showed one of the highest IC_50_ and did not inhibit KLK7.

An interesting observation is that the composition of the formulations somehow affected the antibodies’ activity. For instance, although the amount of A10 in the receptor medium was not significantly different between the formulations (F127 and F127/P123), the inhibitory effect was more pronounced for A10 associated with F127. Similarly, the amount released of C11 from F127/P123 was higher than that obtained from the F127 hydrogel, but the inhibitory effect was more efficient for C11-F127. On the other hand, for D11, the release was improved by adding P123, which reflected an improvement on the inhibitory effect, in comparison to the F127 formulation.

In general, all antibodies present similar structural properties (molecular weight, pI, etc.). However, the main differences are observed regarding the 29.6% cysteine in the B10 amino acid composition when compared to A10 (28.6%), C11 (27.7%), and D11 (27.6%) (Supporting Information). A11, C11, and D11 exhibited lower IC_50_ values representing the best enzyme affinity profiles, which were conserved even after incorporation into hydrogels, as observed by data from inhibitory residual activity at 24 h (Figure 4). On the other hand, differential activity profiles were observed after incorporation into F127 or F127/P123. Our hypothesis to explain these results involves interactions between the PL molecules and the exposure of essential binding/recognition regions between the antibodies and the target enzyme site, which modulate the antibody activity.^62^ In this sense, molecular dynamics studies are in course to clarify the interactions between antibody binding regions and polymer micelles.

### 3.4 *In Vitro* Permeation Assays: Artificial Membranes and Porcine Ear Skin

**Figure 5:**
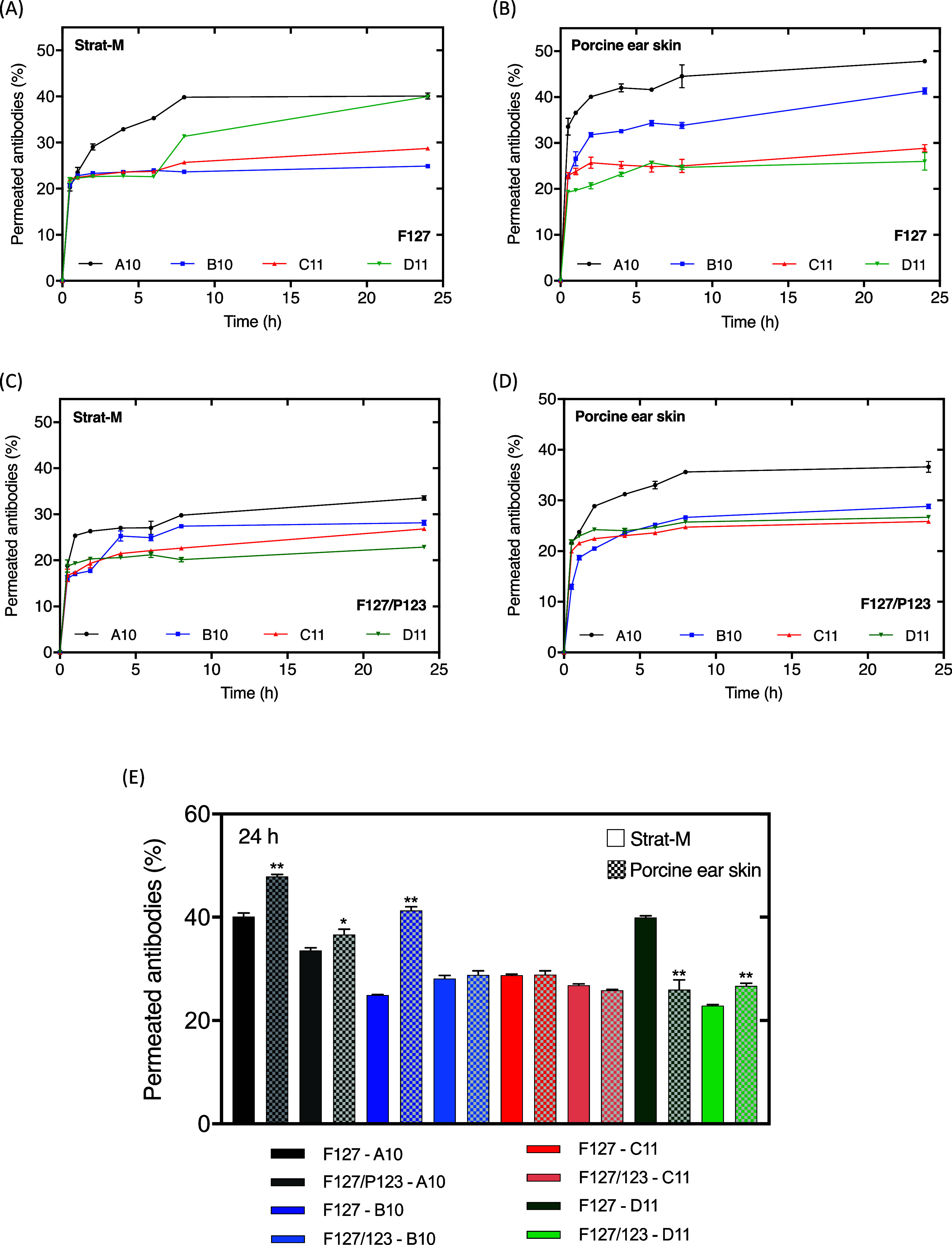
Permeation profiles of antibodies A10, B10, C11, and D11 in Strat-M (A,C) and porcine ear skin (B,D) encapsulated in Pluronic F127 30% w/v (A,B) and F127/P123 28–2% w/v (C,D). Comparison graph between the maximum amount permeated of the antibodies (E) using the Strat-M membrane and porcine ear skin. Strat-M vs porcine ear skin permeation (*p* < 0.0001 [**] and *p* < 0.0008 [*]) by ANOVA (one-way)–Tukey’s multiple comparison tests (*n* = 6).

*In vitro* permeation assays were designed to access the permeability of scFv-Fcs through the skin. Dermatomed porcine ear skin and a Strat-M synthetic membrane were used to perform these experiments. Although permeation is desired for most dermatological applications, for this study, the antibodies must remain on the outer skin layers since KLK7 is found at the outermost layers of the epidermis, including in the stratum corneum.^63,64^Figure 5 shows the results of permeation assays for Strat-M (A,C) and porcine ear skin (B,D).

In general, all hydrogels showed a controlled antibody permeation profile over the experimental time for both membrane types (Figure 5). However, for F127 gels, the final percentage reached higher values when compared to F127/P123 formulations, mainly for A10 and D11 antibodies (Figure 5E). This observation can be related to the ability of the P123 to play a role in possible antibody retention into the hydrogel’s formulation.

Compared to small molecules like budesonide (an anti-inflammatory glucocorticoid), the addition of P123 promoted the opposite effect, i.e., reducing the drug’s skin accumulation in the porcine epidermis.^38^ It is worth mentioning that the permeation ability of the antibodies was unexpectedly high (between ∼23 and 41%/24 h), considering their molecular weight (∼30–50 kDa). Kopecki and collaborators investigated the topical permeability of mouse monoclonal antibodies through an *in vivo* approach.^65^ The authors reported that when incorporated into a cream vehicle and applied topically to the skin, the antibodies successfully penetrated the basal epidermis and upper dermis. These results support the idea that massive antibodies, such as monoclonal antibodies, can reach dermis layers when applied topically. Due to the hydrophobic and compact characteristics of the stratum corneum, the use of low-molecular-weight (<500 Da) molecules with moderate lipophilicity is preferable when the objective is to permeate the skin.^66^ Nonetheless, the physicochemical proprieties of the bioactives are not the only modulators of the permeation efficiency; the formulation composition also contributes significantly to the permeation profile.

PLs are well-known as excellent excipients for topical applications, promoting and controlling the permeation profiles of various types of drugs through the skin.^67^ From the results presented in this work, possible further modifications in the composition could be investigated to improve the retention of these antibodies in the upper layers of the epidermis. For instance, an oil phase with high affinity with the stratum corneum lipid matrix may be added to the hydrogels in an attempt to enhance the bioactive retention.^68,69^

Comparing the permeation with the release results, the permeation presented a more gradual release pattern over time, which is expected due to the complexity of the membranes and the possibility of their interaction with the formulations. The antibody permeation is a complex process that includes the release from the gel and the penetration/diffusion into the stratum corneum until the target site is reached. These processes are more representative in the permeation assay using a human skin mimetic membrane or an *ex vivo* skin model. In this context, the behavior of the permeation curves for the antibodies was similar between the Strat-M membrane and the porcine ear skin, demonstrating these model’s potential in early stages of the pharmaceutical development.

Until now, the discussion was about the amount of antibodies that have permeated the membranes. However, the theoretical amount of antibodies that remained accumulated within the membranes will be the fraction available to inhibit KLK7. The theoretical amount retained in porcine skin for the A10, C11, and D11 antibodies was around 20–30% below their IC_50_. These quantities should be enough to show a partial inhibition of KLK7, which can control its activity but not completely inhibit the enzyme (according to previously calculated IC_50_ values).^30^

Herein, our goal is to deliver the antibodies into the skin’s most superficial layer; they must keep showing the desired biological activity after being encapsulated into the hydrogels. Considering that clinical manifestations of NS (scaling and atopic skin) result from a mutation into the encoding specific inhibitor (LEKTI), the highly exposed skin damage would allow either the permeability or the maintenance of active scFv-Fcs released from the micellar hydrogels directly into the site of action. This possible delivery mechanism, favored by inherent pathological manifestations, could provide a new strategy for the treatment of NS. Another important point is that the KLK 5 or 7 inhibition could avoid the clinical manifestations of the mutated LEKTI, as observed by Kasparek et al.^14^ when they described the benefits of regenerating the cutaneous barrier with no side effects, after KLK5 and KLK7 inhibition. Then, from a clinical perspective, the partial inhibition of KLK7 can be also beneficial since the protein functions into the tissue, i.e., it maintains the barrier homeostasis by regulating the cleavage of corneodesmosomes.^70^

## 4 Conclusions

After the generation of four novel scFv-Fcs with high inhibitory effects against kallikrein 7 (KLK7) using phage display technology by our group, this work proposed encapsulation of these antibodies into Pluronic (PL)-based systems. To the best of our knowledge, this is the first proposal of topical formulations based on PL hydrogels to carry scFv-Fc antibodies. Hydrogels composed of F127 30% w/v and F127/P123 28–2% w/v and loaded with scFv-Fcs antibodies (A10, B10, C11, and D11) were characterized by their physiochemical properties. The formulations containing PL P123 showed improved viscoelastic properties and a gelation temperature of around 20 °C. Moreover, all formulations presented *T*_sol–gel_ below the skin temperature, high storage modulus (*G*′) contribution, and pseudoplasticity. These properties are advantageous for topical applications due to preventing phase separation and facilitating spreadability. After 24 h of permeation evaluation using a Strat-M synthetic membrane and porcine ear skin, the antibodies showed controlled-release patterns and presented inhibitory activity against KLK7. In summary, this work demonstrated potential agents targeting KLK7 that can provide new strategies for treating atopic dermatitis and other diseases related to skin desquamation. Future studies should be conducted to improve the retention of the antibodies in the upper layers of the epidermis as well as to evaluate the inhibition of KLK7 at the target site into the skin.

## Supporting Information Available

Antibody sequences and nomenclature, antibody *in vitro* release modeling, and scFv-Fc antibody amino acid compositions (PDF)

## References

[ref1] Mullard A. 2018 FDA drug approvals. Nature reviews. Drug discovery 2019, 18, 85–89. 10.1038/d41573-019-00014-x. 30710142

[ref2] Shim H. Therapeutic Antibodies by Phage Display. Current pharmaceutical design. 2017, 22, 6538–6559. 10.2174/1381612822666160923113714. 27669967

[ref3] Lonberg N. Human monoclonal antibodies from transgenic mice. Handbook of experimental pharmacology. 2008, 181, 69–97. 10.1007/978-3-540-73259-4_4. 18071942 PMC7120671

[ref4] Bradbury A. R. M.; Sidhu S.; Dübel S.; McCafferty J. Beyond natural antibodies: the power of in vitro display technologies. Nature biotechnology. 2011, 29, 245–254. 10.1038/nbt.1791. PMC3057417 21390033

[ref5] Schirrmann T.; Meyer T.; Schütte M.; Frenzel A.; Hust M. Phage display for the generation of antibodies for proteome research, diagnostics and therapy. Molecules (Basel, Switzerland). 2011, 16, 412–426. 10.3390/molecules16010412. 21221060 PMC6259421

[ref6] Frenzel A.; Hust M.; Schirrmann T. Expression of recombinant antibodies. Front. Immunol. 2013, 4, 217 10.3389/fimmu.2013.00217. 23908655 PMC3725456

[ref7] Bitoun E.; Chavanas S.; Irvine A. D.; Lonie L.; Bodemer C.; Paradisi M.; Hamel-Teillac D.; Ansai S.; Mitsuhashi Y.; Taïeb A.; de Prost Y.; Zambruno G.; Harper J. I.; Hovnanian A. Netherton syndrome: disease expression and spectrum of SPINK5 mutations in 21 families. Journal of investigative dermatology. 2002, 118, 352–361. 10.1046/j.1523-1747.2002.01603.x. 11841556

[ref8] Shaw J. L. V.; Diamandis E. P. Distribution of 15 human kallikreins in tissues and biological fluids. Clinical Chemistry. 2007, 53, 1423–1432. 10.1373/clinchem.2007.088104. 17573418

[ref9] Stefanini A. C. B.; Da Cunha B. R.; Henrique T.; Tajara E. H. Involvement of kallikrein-related peptidases in normal and pathologic processes. Dis. Markers 2015, 2015, 946572 10.1155/2015/946572. 26783378 PMC4689925

[ref10] Pampalakis G.; Sotiropoulou G. Tissue kallikrein proteolytic cascade pathways in normal physiology and cancer. Biochim. Biophys. Acta 2007, 1776, 22–31. 10.1016/j.bbcan.2007.06.001. 17629406

[ref11] Sotiropoulou G.; Zingkou E.; Pampalakis G. Reconstructing the epidermal proteolytic cascades in health and disease. J. Pathol. 2022, 257, 545–560. 10.1002/path.5888. 35218558

[ref12] Jonca N.; Leclerc E. A.; Caubet C.; Simon M.; Guerrin M.; Serre G. Corneodesmosomes and corneodesmosin: from the stratum corneum cohesion to the pathophysiology of genodermatoses. Eur. J. Dermatol. 2011, 21 (Suppl 2), 35–42. 10.1684/ejd.2011.1264. 21628128

[ref13] Bisyris E.; Zingkou E.; Kordopati G. E.; Matsoukas M.; Magriotis P. A.; Pampalakis G.; Sotiropoulou G. A novel theranostic activity-based probe targeting kallikrein 7 for the diagnosis and treatment of skin diseases. Chem. Commun. (Camb). 2021, 57, 6507–6510. 10.1039/D1CC01673C. 34105530

[ref14] Kasparek P.; Ileninova Z.; Zbodakova O.; Kanchev I.; Benada O.; Chalupsky K.; Brattsand M.; Beck I. M.; Sedlacek R. KLK5 and KLK7 Ablation Fully Rescues Lethality of Netherton Syndrome-Like Phenotype. PLoS Genet. 2017, 13, e1006566 10.1371/journal.pgen.1006566. 28095415 PMC5283769

[ref15] Hubiche T.; Ged C.; Benard A.; Léauté-Labrèze C.; McElreavey K.; De Verneuil H.; Taïeb A.; Boralevi F. Analysis of SPINK 5, KLK 7 and FLG genotypes in a French atopic dermatitis cohort. Acta Dermato-Venereologica 2007, 87, 499–505. 10.2340/00015555-0329. 17989887

[ref16] Yamasaki K.; Schauber J.; Coda A.; Lin H.; Dorschner R. A.; Schechter N. M.; Bonnart C.; Descargues P.; Hovnanian A.; Gallo R. L. Kallikrein-mediated proteolysis regulates the antimicrobial effects of cathelicidins in skin. FASEB Journal. 2006, 20, 2068–2080. 10.1096/fj.06-6075com. 17012259

[ref17] Martins W. K.; Esteves G. H.; Almeida O. M.; Rezze G. G.; Landman G.; Marques S. M.; Carvalho A. F.; Reis L. F. L.; Duprat J. P.; Stolf B. S. Gene network analyses point to the importance of human tissue kallikreins in melanoma progression. BMC Med. Genomics 2011, 4, 76 10.1186/1755-8794-4-76. 22032772 PMC3212933

[ref18] Deraison C.; Bonnart C.; Lopez F.; Besson C.; Robinson R.; Jayakumar A.; Wagberg F.; Brattsand M.; Hachem J. P.; Leonardsson G.; Hovnanian A. LEKTI fragments specifically inhibit KLK5, KLK7, and KLK14 and control desquamation through a pH-dependent interaction. Mol. Biol. Cell 2007, 18, 3607–3619. 10.1091/mbc.e07-02-0124. 17596512 PMC1951746

[ref19] Furio L.; Pampalakis G.; Michael I. P.; Nagy A.; Sotiropoulou G.; Hovnanian A. KLK5 Inactivation Reverses Cutaneous Hallmarks of Netherton Syndrome. PLoS Genet. 2015, 11, e1005389 10.1371/journal.pgen.1005389. 26390218 PMC4577096

[ref20] Jendrny C.; Beck-Sickinger A. G. Inhibition of Kallikrein-Related Peptidases 7 and 5 by Grafting Serpin Reactive-Center Loop Sequences onto Sunflower Trypsin Inhibitor-1 (SFTI-1). Chembiochem: a European Journal of Chemical Biology. 2016, 17, 719–726. 10.1002/cbic.201500539. 26574674

[ref21] de Veer S. J.; Ukolova S. S.; Munro C. A.; Swedberg J. E.; Buckle A. M.; Harris J. M. Mechanism-based selection of a potent kallikrein-related peptidase 7 inhibitor from a versatile library based on the sunflower trypsin inhibitor SFTI-1. Biopolymers. 2013, 100, 510–518. 10.1002/bip.22231. 24078181

[ref22] de Veer S. J.; Furio L.; Swedberg J. E.; Munro C. A.; Brattsand M.; Clements J. A.; Hovnanian A.; Harris J. A. Selective substrates and inhibitors for kallikrein-related peptidase 7 (KLK7) shed light on KLK proteolytic activity in the stratum corneum. J. Invest Dermatol 2017, 137, 430–439. 10.1016/j.jid.2016.09.017. 27697464

[ref23] Chavarria-Smith J.; Chiu C. P. C.; Jackman J. K.; Yin J.; Zhang J.; Hackney J. A.; Lin W. Y.; Tyagi T.; Sun Y.; Tao J.; Dunlap D.; Morton W. D.; Ghodge S. V.; Maun H. R.; Li H.; Hernandez-Barry H.; Loyet K. M.; Chen E.; Liu J.; Tam C.; Yaspan B. L.; Cai H.; Balazs M.; Arron J. R.; Li J.; Wittwer A. J.; Pappu R.; Austin C. D.; Lee W. P.; Lazarus R. A.; Sudhamsu J.; Koerber J. T.; Yi T. Dual antibody inhibition of KLK5 and KLK7 in Netherton syndrome and atopic dermatitis. Sci. Transl. Med. 2022, 14, eabp9159 10.1126/scitranslmed.abp9159. 36516271

[ref24] Teixeira T. S. P.; Freitas R. F.; Abrahão O. J.; Devienne K. F.; de Souza L. R.; Blaber S. I.; Blaber M.; Kondo M. Y.; Juliano M. A.; Juliano L.; Puzer L. Biological evaluation and docking studies of natural isocoumarins as inhibitors for human kallikrein 5 and 7. Bioorganic & medicinal chemistry letters. 2011, 21, 6112–6115. 10.1016/j.bmcl.2011.08.044. 21903387

[ref25] Santos J. A. N.; Kondo M. Y.; Freitas R. F.; dos Santos M. H.; Ramalho T. C.; Assis D. M.; Juliano L.; Juliano M. A.; Puzer L. The natural flavone fukugetin as a mixed-type inhibitor for human tissue kallikreins. Bioorganic & medicinal chemistry letters. 2016, 26, 1485–1489. 10.1016/j.bmcl.2016.01.039. 26848109

[ref26] Freitas R. F.; Teixeira T. S. P.; Barros T. G.; Santos J. A. N.; Kondo M. Y.; Juliano M. A.; Juliano L.; Blaber M.; Antunes O. A. C.; Abrahão O. J.; Pinheiro S.; Muri E. M. F.; Puzer L. Isomannide derivatives as new class of inhibitors for human kallikrein 7. Bioorganic & medicinal chemistry letters. 2012, 22, 6072–6075. 10.1016/j.bmcl.2012.08.047. 22959247

[ref27] Oliveira J. P. C.; Freitas R. F.; Melo L. S. de; Barros T. G.; Santos J. A. N.; Juliano M. A.; Pinheiro S.; Blaber M.; Juliano L.; Muri E. M. F.; Puzer L. Isomannide-based peptidomimetics as inhibitors for human tissue kallikreins 5 and 7. ACS medicinal chemistry letters. 2014, 5, 128–132. 10.1021/ml4003698. 24900785 PMC4027626

[ref28] de Souza A. S.; Pacheco B. D. C.; Pinheiro S.; Muri E. M. F.; Dias L. R. S.; Lima C. H. S.; Garrett R.; de Moraes M. B. M.; de Souza B. E. G.; Puzer L. 3-Acyltetramic acids as a novel class of inhibitors for human kallikreins 5 and 7. Bioorganic & medicinal chemistry letters. 2019, 29, 1094–1098. 10.1016/j.bmcl.2019.02.031. 30833107

[ref29] Barros T. G.; Santos J. A. N.; de Souza B. E. G.; Sodero A. C. R.; de Souza A. M. T.; da Silva D. P.; Rodrigues C. R.; Pinheiro S.; Dias L. R. S.; Abrahim-Vieira B.; Puzer L.; Muri E. M. F. Discovery of a new isomannide-based peptidomimetic synthetized by Ugi multicomponent reaction as human tissue kallikrein 1 inhibitor. Bioorganic & medicinal chemistry letters. 2017, 27, 314–318. 10.1016/j.bmcl.2016.11.051. 27914800

[ref30] Laureano A. F. S.; Zani M. B.; Sant’Ana A. M.; Tognato R. C.; Lombello C. B.; do Nascimento M. H. M.; Helmsing S.; Fühner V.; Hust M.; Puzer L. Generation of recombinant antibodies against human tissue kallikrein 7 to treat skin diseases. Bioorg. Med. Chem. Lett. 2020, 30, 127626 10.1016/j.bmcl.2020.127626. 33096161

[ref31] Aguilar-Toala J. E.; Quintanar-Guerrero D.; Liceaga A. M.; Zambrano- Zaragoza M. L. Encapsulation of bioactive peptides: A strategy to improve the stability, protect the nutraceutical bioactivity and support their food applications. RSC Advances. 2022, 12, 6449–6458. 10.1039/D1RA08590E. 35424621 PMC8982217

[ref32] Tibbitt M. W.; Dahlman J. E.; Langer R. Emerging Frontiers in Drug Delivery. J. Am. Chem. Soc. 2016, 138, 704–717. 10.1021/jacs.5b09974. 26741786

[ref33] Paolino D.; Sinha P.; Fresta M.; Ferrari M.; Webster J. G. Principles of Controled Drug Delivery. In Encyclopedia of Medical Devices and Instrumentation; Wiley: 2006; 437-495DOI: 10.1002/0471732877.emd274.

[ref34] Bodratti A. M.; Alexandridis P. Formulation of poloxamers for drug delivery. Journal of Functional Biomaterials. 2018, 9, 11 10.3390/jfb9010011. 29346330 PMC5872097

[ref35] Pitto-Barry A.; Barry N. P. E. Pluronic® block-copolymers in medicine: From chemical and biological versatility to rationalisation and clinical advances. Polymer Chemistry. 2014, 5, 3291–3297. 10.1039/C4PY00039K.

[ref36] Gioffredi E.; Boffito M.; Calzone S.; Giannitelli S. M.; Rainer A.; Trombetta M.; Mozetic P.; Chiono V. Pluronic F127 Hydrogel Characterization and Biofabrication in Cellularized Constructs for Tissue Engineering Applications. Procedia CIRP. 2016, 49, 125–132. 10.1016/j.procir.2015.11.001.

[ref37] Shaarani S.; Hamid S. S.; Kaus N. H. M. The Influence of pluronic F68 and F127 nanocarrier on physicochemical properties, in vitro release, and antiproliferative activity of thymoquinone drug. Pharmacogn. Res. 2017, 9, 12–20. 10.4103/0974-8490.199774. PMC5330097 28250648

[ref38] Padula C.; Machado I. P.; Vigato A. A.; De Araujo D. R. New strategies for improving budesonide skin retention. Pharmaceutics 2022, 14, 30 10.3390/pharmaceutics14010030. PMC8781796 35056927

[ref39] Grillo R.; Dias F. V.; Querobino S. M.; Alberto-Silva C.; Fraceto L. F.; de Paula E.; de Araujo D. R. Influence of hybrid polymeric nanoparticle/thermosensitive hydrogels systems on formulation tracking and in vitro artificial membrane permeation: A promising system for skin drug-delivery. Colloids and Surfaces B: Biointerfaces. 2019, 174, 56–62. 10.1016/j.colsurfb.2018.10.063. 30439638

[ref40] Escobar-Chávez J. J.; Quintanar-Guerrero D.; Ganem-Quintanar A. In vivo skin permeation of sodium naproxen formulated in pluronic F-127 gels: Effect of Azone® and Transcutol®. Drug Dev. Ind. Pharm. 2005, 31, 447–454. 10.1080/03639040500214662. 16093210

[ref41] De Souza L. R.; Melo P. M.; Paschoalin T.; Carmona A. K.; Kondo M.; Hirata I. Y.; Blaber M.; Tersariol I.; Takatsuka J.; Juliano M. A.; Juliano L.; Gomes R. A.; Puzer L. Human tissue kallikreins 3 and 5 can act as plasminogen activator releasing active plasmin. Biochem. Biophys. Res. Commun. 2013, 433, 333–337. 10.1016/j.bbrc.2013.03.001. 23500465

[ref42] Schmolka I. R. Artificial skin I. Preparation and properties of pluronic F-127 gels for treatment of burns. Journal of Biomedical Materials Research. 1972, 6, 571–582. 10.1002/jbm.820060609. 4642986

[ref43] Santos A. C. S.; Campos E. V. R.; Keppler A. F.; Fraceto L. F.; De Paula E.; Tófoli G. R.; De Araujo D. R. Budesonide-hydroxypropyl-β-cyclodextrin inclusion complex in binary poloxamer 407/403 system for ulcerative colitis treatment: A physico-chemical study from micelles to hydrogels. Colloids Surf., B 2016, 138, 138–147. 10.1016/j.colsurfb.2015.11.048. 26674842

[ref44] Vigato A. A.; Querobino A. M.; de Faria N. C.; de Freitas A. C. P.; Leonardi G. R.; de Paula E.; Cereda C. M. S.; Tófoli G. R.; de Araujo D. R. Synthesis and characterization of nanostructured lipid-poloxamer organogels for enhanced skin local anesthesia. European Journal of Pharmaceutical Sciences. 2019, 128, 270–278. 10.1016/j.ejps.2018.12.009. 30553060

[ref45] Kushan E.; Senses E. Thermoresponsive and Injectable Composite Hydrogels of Cellulose Nanocrystals and Pluronic F127. ACS Applied Bio Materials. 2021, 4, 3507–3517. 10.1021/acsabm.1c00046. 35014435

[ref46] Zhang Y.; Lam Y. M.; Tan W. S. Poly(ethylene oxide)-poly(propylene oxide)-poly(ethylene oxide)-g-poly(vinylpyrrolidone): association behavior in aqueous solution and interaction with anionic surfactants. Journal of colloid and interface science. 2005, 285, 74–79. 10.1016/j.jcis.2004.12.033. 15797398

[ref47] Akkari A. C. S.; Papini J. Z. B.; Garcia G. K.; Franco M. K. K. D.; Cavalcanti L. P.; Gasperini A.; Alkschbirs M. I.; Yokaichyia F.; De Paula E.; Tófoli G. R.; De Araujo D. R. Poloxamer 407/188 binary thermosensitive hydrogels as delivery systems for infiltrative local anesthesia: Physico-chemical characterization and pharmacological evaluation. Materials Science and Engineering C 2016, 68, 299–307. 10.1016/j.msec.2016.05.088. 27524024

[ref48] Nascimento M. H. M.; Franco M. K. K. D.; Yokaichyia F.; de Paula E.; Lombello C. B.; de Araujo D. R. Hyaluronic acid in Pluronic F-127/F-108 hydrogels for postoperative pain in arthroplasties: Influence on physico-chemical properties and structural requirements for sustained drug-release. International Journal of Biological Macromolecules. 2018, 111, 1245–1254. 10.1016/j.ijbiomac.2018.01.064. 29339288

[ref49] Dos Santos A. C. M.; Akkari A. C. S.; Ferreira I. R. S.; Maruyama C. R.; Pascoli M.; Guilherme V. A.; De Paula E.; Fraceto L. F.; De Lima R.; Melo P. D. S.; De Araujo D. R. Poloxamer-based binary hydrogels for delivering tramadol hydrochloride: Sol-gel transition studies, dissolution-release kinetics, in vitro toxicity, and pharmacological evaluation. Int. J. Nanomed. 2015, 10, 2391–2401. 10.2147/IJN.S72337. PMC4381629 25848258

[ref50] Sun Y.; Wang Q.; Chen J.; Liu L.; Ding L.; Shen M.; Li J.; Han B.; Duan Y. Temperature-sensitive gold nanoparticle-coated pluronic-PLL nanoparticles for drug delivery and chemo-photothermal therapy. Theranostics 2017, 7, 4424–4444. 10.7150/thno.18832. 29158837 PMC5695141

[ref51] Ganguly R.; Kumar S.; Tripathi A.; Basu M.; Verma G.; Sarma H. D.; Chaudhari D. P.; Aswal V. K.; Melo J. S. Structural and therapeutic properties of Pluronic® P123/F127 micellar systems and their modulation by salt and essential oil. J. Mol. Liq. 2020, 310, 113231 10.1016/j.molliq.2020.113231.

[ref52] Russo A.; Pellosi D. S.; Pagliara V.; Milone M. R.; Pucci B.; Caetano W.; Hioka N.; Budillon A.; Ungaro F.; Russo G.; Quaglia F. Biotin-targeted Pluronic® P123/F127 mixed micelles delivering niclosamide: A repositioning strategy to treat drug-resistant lung cancer cells. Int. J. Pharm. 2016, 511, 127–139. 10.1016/j.ijpharm.2016.06.118. 27374195

[ref53] Sepulveda A. F.; Kumpgdee-Vollrath M.; Franco M. K. K. D.; Yokaichiya F.; de Araujo D. R. Supramolecular structure organization and rheological properties modulate the performance of hyaluronic acid-loaded thermosensitive hydrogels as drug-delivery systems. J. Colloid Interface Sci. 2023, 630, 328–340. 10.1016/j.jcis.2022.10.064. 36327735

[ref54] Salay L. C.; Prazeres E. A.; Marín Huachaca N. S.; Lemos M.; Piccoli J. P.; Sanches P. R. S.; Cilli E. M.; Santos R. S.; Feitosa E. Molecular interactions between Pluronic F127 and the peptide tritrpticin in aqueous solution. Colloid Polym. Sci. 2018, 296, 809–817. 10.1007/s00396-018-4304-0.

[ref55] Burak J.; Grela K. P.; Pluta J.; Karolewicz B.; Marciniak D. M. Impact of sterilisation conditions on the rheological properties of thermoresponsive pluronic F-127-based gels for the ophthalmic use. Acta Poloniae Pharmaceutica - Drug Research. 2018, 75, 471–481. 10.32383/appdr/146102.

[ref56] Martinez R. M.; Rosado C.; Velasco M. V. R.; Lannes S. C. S.; Baby A. R. Main features and applications of organogels in cosmetics. International Journal of Cosmetic Science. 2019, 41, 109–117. 10.1111/ics.12519. 30994939

[ref57] Lee C. M.; Jin S. P.; Doh E. J.; Lee D. H.; Chung J. H. Regional variation of human skin surface temperature. Annals of Dermatology. 2019, 31, 349–352. 10.5021/ad.2019.31.3.349. 33911607 PMC7992731

[ref58] Safdari Y.; Ahmadzadeh V.; Khalili M.; Jaliani H. Z.; Zarei V.; Erfani-Moghadam V. Use of single-chain antibody derivatives for targeted drug delivery. Mol. Med. 2016, 22, 258–270. 10.2119/molmed.2016.00043. 27249008 PMC5023511

[ref59] Lim K. J.; Sung B. H.; Shin J. R.; Lee Y. W.; Kim D. J.; Yang K. S.; Kim S. C. A cancer specific cell-penetrating peptide, BR2, for the efficient delivery of an scFv into cancer cells. PloS one. 2013, 8, e66084 10.1371/journal.pone.0066084. 23776609 PMC3679022

[ref60] Choi J. H.; Joung Y. K.; Bae J. W.; Choi J. W.; Quyen T. N.; Park K. D. Self-assembled nanogel of pluronic-conjugated heparin as a versatile drug nanocarrier. Macromolecular Research. 2011, 19, 180–188. 10.1007/s13233-011-0214-4.

[ref61] Haidari H.; Zhang Q.; Melville E.; Kopecki Z.; Song Y.; Cowin A. J.; Garg S. Development of Topical Delivery Systems for Flightless Neutralizing Antibody. J. Pharm. Sci. 2017, 106, 1795–1804. 10.1016/j.xphs.2017.03.012. 28336300

[ref62] Soshee A.; Zürcher S.; Spencer N. D.; Halperin A.; Nizak C. General in vitro method to analyze the interactions of synthetic polymers with human antibody repertoires. Biomacromolecules. 2014, 15, 113–121. 10.1021/bm401360y. 24328191

[ref63] Delaunay T.; Deschamps L.; Haddada M.; Walker F.; Soosaipillai A.; Soualmia F.; El Amri C.; Diamandis E. P.; Brattsand M.; Magdolen V.; Darmoul D. Aberrant expression of kallikrein-related peptidase 7 is correlated with human melanoma aggressiveness by stimulating cell migration and invasion. Molecular oncology. 2017, 11, 1330–1347. 10.1002/1878-0261.12103. 28636767 PMC5623816

[ref64] Igawa S.; Kishibe M.; Minami-Hori M.; Honma M.; Tsujimura H.; Ishikawa J.; Fujimura T.; Murakami M.; Ishida-Yamamoto A. Incomplete KLK7 Secretion and Upregulated LEKTI Expression Underlie Hyperkeratotic Stratum Corneum in Atopic Dermatitis. Journal of Investigative Dermatology. 2017, 137, 449–456. 10.1016/j.jid.2016.10.015. 27769847

[ref65] Kopecki Z.; Ruzehaji N.; Turner C.; Iwata H.; Ludwig R. J.; Zillikens D.; Murrell D. F.; Cowin A. J. Topically applied flightless i neutralizing antibodies improve healing of blistered skin in a murine model of epidermolysis bullosa acquisita. Journal of Investigative Dermatology. 2013, 133, 1008–1016. 10.1038/jid.2012.457. 23223144

[ref66] Benson H. A. E.; Namjoshi S. Proteins and peptides: Strategies for delivery to and across the skin. J. Pharm. Sci. 2008, 97, 3591–3610. 10.1002/jps.21277. 18200531

[ref67] Almeida H.; Amaral M. H.; Lobão P.; Lobo J. M. S. Pluronic® F-127 and Pluronic Lecithin Organogel (PLO): main features and their applications in topical and transdermal administration of drugs. Journal of pharmacy & pharmaceutical sciences: a publication of the Canadian Society for Pharmaceutical Sciences, Société canadienne des sciences pharmaceutiques. 2012, 15, 592–605. 10.18433/J3HW2B. 23106961

[ref68] Vigato A. A.; Machado I. P.; Del Valle M.; Da Ana P. A.; Sepulveda A. F.; Yokaichiya F.; Franco M. K. K. D.; Loiola M. C.; Tófoli G. R.; Cereda C. M. S.; De Sairre M. I.; De Araujo D. R. Monoketonic Curcuminoid-Lidocaine Co-Deliver Using Thermosensitive Organogels: From Drug Synthesis to Epidermis Structural Studies. Pharmaceutics 2022, 14, 293 10.3390/pharmaceutics14020293. 35214026 PMC8879257

[ref69] Vigato A. A.; Querobino S. M.; De Faria N. C.; Candido A. C. B. B.; Magalhães L. G.; Cereda C. M. S.; Tófoli G. R.; Campos E. V. R.; Machado I. P.; Fraceto L. F.; De Sairre M. I.; De Araujo D. R. Physico-chemical characterization and biopharmaceutical evaluation of lipid-poloxamer-based organogels for curcumin skin delivery. Front. Pharmacol. 2019, 10, 1006 10.3389/fphar.2019.01006. 31572185 PMC6751402

[ref70] Guo C. J.; Mack M. R.; Oetjen L. K.; Trier A. M.; Council M. L.; Pavel A. B.; Guttman-Yassky E.; Kim B. S.; Liu Q. Kallikrein 7 Promotes Atopic Dermatitis-Associated Itch Independently of Skin Inflammation. J. Invest. Dermatol. 2020, 140, 1244–1252. 10.1016/j.jid.2019.10.022. 31883963 PMC7247952

