# Supplementary material for: Recombinant scFv-Fc Anti-kallikrein 7 Antibody-Loaded Thermosensitive Hydrogels Against Skin Desquamation Disorders
Source: ACS Appl Bio Mater. 2024 Jun 18;7(7):4486–96. doi: 10.1021/acsabm.4c00371 (PMC11253099; doi:10.1021/acsabm.4c00371)
Supplement: Supplementary file 1 — mt4c00371_si_001.pdf [file mt4c00371_si_001.pdf]

SUPPORTING INFORMATION

Recombinant scfv-Fc anti-kallikrein 7 antibodies-loaded thermosensitive hydrogels against skin desquamation disorders

Ana Flávia Santarine Laureano[1,2], Aryane Alves Vigato[3,4], Luciano Puzer[2], Daniele Ribeiro de Araujo[5,6]*

Ana Flávia Santarine Laureano[1,2], Aryane Alves Vigato[3,4], Luciano Puzer[5], Daniele Ribeiro de Araujo[6,7]*

[1] Massachusetts General Hospital & Harvard Medical School – Department of Surgery, Center for transplantation sciences, CNY149 13th Street, Charlestown, MA 02129, Boston, United States of America.

[2] Universidade Federal do ABC, Centro de Ciências Naturais e Humanas, Al. da Universidade, s/n - Anchieta, São Bernardo do Campo - SP, 09606-045. Brasil.

[3]Biomedical Science, Faculty of Health and Society, Malmö University, 20506 Malmö, Sweden.

[4]Biofilms – Research Center for Biointerfaces, Malmö University, 20506 Malmö, Sweden.

[5] Universidade Federal do ABC, Centro de Ciências Naturais e Humanas, Al. da Universidade, s/n - Anchieta, São Bernardo do Campo - SP, 09606-045. Brasil.

[6]Universidade Federal do ABC, Centro de Ciências Naturais e Humanas, Av. dos Estados, 5001. Bloco A, Torre 3. Santo André-SP, 09210-580. Brasil.

[7]Departamento de Biofísica, Escola Paulista de Medicina, Universidade Federal de São Paulo. Rua Botucatu, 862, Vila Clementino. Sao Paulo-SP, 04023-062. Brazil.

ORCID:

Ana Flávia Santarine Laureano: 0000-0002-3521-0580

Aryane Alves Vigato: 0000-0001-8929-8568

Luciano Puzer: 0000-0002-4412-0974

Daniele Ribeiro de Araujo: 0000-0002-9289-4229

*Corresponding authors

Dr. Luciano Puzer - luciano.puzer@ufabc.edu.br

Dr. Daniele Ribeiro Araujo – daniele.ribeiro@unifesp.br or draraujo2012@gmail.com

1. **Antibodies sequences and nomenclature (underline sequence correspond to the link between the heavy and light chains)**

LUP37-A10 (MW=54.4 kDa)

MAQVQLVQSGAEVKKPGASVKVSCKVSGYTLTELSMHWVRQTPGKGLEWMGGFDPEDGETIYAQ
KFQGRVTMTEDTSTDTAYMELSSLRSEDTAVYYCATQGYSGYDYYFDYWGQGTLVTVSSGSASAP
KLEEGEFSEARVQAVVTQEPSLSVSPGGTVTLSCGLSSGSVSSTHYPSWYQQTPGQAPRTLIYNTNIR
SSGVPDRFSGSKSGTSASLAISGLRSEDEADYYCAAWDDSLSGWVFGGGTKLTVLGQPKAAPSVTLF
PPSSAAASDKTHTCPPCPAPELLGGPSVFLFPPKPKDTLMISRTPEVTCVVVDVSHEDPEVKFNWYVD
GVEVHNAKTKPREEQYNSTYRVVSVLTVLHQDWLNGKEYKCKVSNKALPAPIEKTISKAKGQPREP
QVYTLPPSREEVTKNQVSLTCLVKGFYPSDIAVEWESNGQPENNYKTTPPVLDSDGSFFLYSKLTVD
KSRWQQGNVFSCSVMHEALHNHYTQKSLSLSPGK

LUP37-B10 (MW=54.5 kDa)

MAQVQLVQSGAEVKKPGASVKVSCKVSGYTLTELSMHWVRQTPGKGLEWMGGFDPEDGETIYAQKFQGRV
TMTEDTSTDTAYMELSSLRSEDTAVYYCATQGYSGYDYYFDYWGQGTLVTVSSGSASAP**KLEEGEFSEARV**
QAVLTQPPSASGTPGQRVTISCSGSNSNVGRYTVNWYQQLPGTAPKLLINTNDRRPSGVPDRFSGSKSGTSASL
AISGLQSEDEAAYYCAAWDDSRNGWAFGGGTKLTVLRQPKAAPSVTLFPPSSAAASDKTHTCPPCPAPELLGG
PSVFLFPPKPKDTLMISRTPEVTCVVVDVSHEDPEVKFNWYVDGVEVHNAKTKPREEQYNSTYRVVSVLTVL
HQDWLNGKEYKCKVSNKALPAPIEKTISKAKGQPREPQVYTLPPSREEVTKNQVSLTCLVKGFYPSDIAVEWE
SNGQPENNYKTTPPVLDSDGSFFLYSKLTVDKSRWQQGNVFSCSVMHEALHNHYTQKSLSLSPGK

LUP37-C11 (MW=54.3 kDa)

MAQVQLVQSGAEVKKPGASVKVSCKVSGYTLTELSMHWVRQTPGKGLEWMGGFDPEDGETIYAQKFQGRV
TMTEDTSTDTAYMELSSLRSEDTAVYYCATQGYSGYDYYFDYWGQGTLVTVSSGSASAP**KLEEGEFSEARV**
SSELTQDPAVSVALGQTVRITCQGDSLRNYYASWYQQKPGQAPVVVIYGKNNRPSGIPDRFSGSSSGNTASLTI
TGAQAEDEADYYCNSRDSSGNHVVFGGGTKLTVLGQSKAAPSVTLFPPSSAAASDKTHTCPPCPAPELLGGPS
VFLFPPKPKDTLMISRTPEVTCVVVDVSHEDPEVKFNWYVDGVEVHNAKTKPREEQYNSTYRVVSVLTVLHQ
DWLNGKEYKCKVSNKALPAPIEKTISKAKGQPREPQVYTLPPSREEVTKNQVSLTCLVKGFYPSDIAVEWESN
GQPENNYKTTPPVLDSDGSFFLYSKLTVDKSRWQQGNVFSCSVMHEALHNHYTQKSLSLSPGK

LUP37-D11(MW=54.4 kDa)

MAQVQLVQSGAEVKKPGASVKVSCKVSGYTLTELSMHWVRQTPGKGLEWMGGFDPEDGETIYAQKFQGRV
TMTEDTSTDTAYMELSSLRSEDTAVYYCATQGYSGYDYYFDYWGQGTLVTVSSGSASAP**KLEEGEFSEARV**
SSELTQDPAVSVALGQTVRITCQGDSLRSYYASWYQQKPGQAPVVVIYGKNDRPSGIPDRFSGSSSGNTASLTI
TGAQAEDEADYYCNSRDSSGNHHVVFGGGTKLTVLGQPKAAPSVTLFPPSSAAASDKTHTCPPCPAPELLGGP

SVFLFPPKPKDTLMISRTPEVTCVVVDVSHEDPEVKFNWYVDGVEVHNAKTKPREEQYNSTYRVVSVLTVLH QDWLNGKEYKCKVSNKALPAPIEKTISKAKGQPREPQVYTLPPSREEVTKNQVSLTCLVKGFYPSDIAVEWES NGQPENNYKTTPPVLDSDGSFFLYSKLTVDKSRWQQGNVFSCSVMHEALHNHYTQKSLSLSPGK

2. Antibodies in vitro release modelling

Drug release mechanisms were determined by applying following mathematical models: zero-order, Higuchi, and Korsmeyer-Peppas (Costa and Lobo, 2001), as described below (Eqs. 1, 2 and 3, respectively):

$$Q(t) = Q_0 + K_0 t \qquad (Eq. 1)$$

where Q is the of drug released amount at time t, Q_0 is the initial drug concentration, and K_0 is the release constant.

$$Q(t) = K_H \sqrt{t} \qquad (Eq. 2)$$

where K_H is the drug release constant, and

$$\frac{M(t)}{M_\infty} = K_{KP} t^n \qquad (Eq. 3)$$

where $M(t)/M_\infty$ describes the drug released fraction against time t. K_{KP} is the release constant, and n is the exponent. For n values of 0.45, the mechanism is described by Fickian diffusion, while for 0.45 < n < 0.89, is non-Fickian or anomalous transport. Case-II and super case-II transports are considered when n=0.89 and > 0.89, respectively, with potential disturbing into the formulations internal structure by erosion. All results were analyzed by linear regression, and determination coefficient (R^2) calculated.

A10

Number of amino acids: 814

Molecular weight: 67434.61

Theoretical pI: 5.06

Amino acid composition: CSV format

Ala	(A)	174	21.4%
Arg	(R)	0	0.0%
Asn	(N)	0	0.0%
Asp	(D)	0	0.0%
Cys	(C)	233	28.6%
Gln	(Q)	0	0.0%
Glu	(E)	0	0.0%
Gly	(G)	230	28.3%
His	(H)	0	0.0%
Ile	(I)	0	0.0%
Leu	(L)	0	0.0%
Lys	(K)	0	0.0%
Met	(M)	0	0.0%
Phe	(F)	0	0.0%
Pro	(P)	0	0.0%
Ser	(S)	0	0.0%
Thr	(T)	177	21.7%
Trp	(W)	0	0.0%
Tyr	(Y)	0	0.0%
Val	(V)	0	0.0%
Pyl	(O)	0	0.0%
Sec	(U)	0	0.0%
	(B)	0	0.0%
	(Z)	0	0.0%
	(X)	0	0.0%

Total number of negatively charged residues (Asp + Glu): 0
Total number of positively charged residues (Arg + Lys): 0

Atomic composition:

Carbon	C	2389
Hydrogen	H	3966
Nitrogen	N	814
Oxygen	O	992
Sulfur	S	233

Formula: $C_{2389}H_{3966}N_{814}O_{992}S_{233}$
Total number of atoms: 8394

Extinction coefficients:

This protein does not contain any Trp residues. Experience shows that this could result in more than 10% error in the computed extinction coefficient.

Extinction coefficients are in units of M^{-1} cm^{-1}, at 280 nm measured in water.

Ext. coefficient 14500
Abs 0.1% (=1 g/l) 0.215, assuming all pairs of Cys residues form cystines

Ext. coefficient 0
Abs 0.1% (=1 g/l) 0.000, assuming all Cys residues are reduced

Estimated half-life:

The N-terminal of the sequence considered is C (Cys).

The estimated half-life is: 1.2 hours (mammalian reticulocytes, in vitro).
>20 hours (yeast, in vivo).
>10 hours (Escherichia coli, in vivo).

Instability index:

The instability index (II) is computed to be 58.14
This classifies the protein as unstable.

Aliphatic index: 21.38

Grand average of hydropathicity (GRAVY): 0.835

B10

Number of amino acids: 810

Molecular weight: 67014.40

Theoretical pI: 5.05

Amino acid composition: CSV format

Ala	(A)	175	21.6%
Arg	(R)	0	0.0%
Asn	(N)	0	0.0%
Asp	(D)	0	0.0%
Cys	(C)	240	29.6%
Gln	(Q)	0	0.0%
Glu	(E)	0	0.0%
Gly	(G)	230	28.4%
His	(H)	0	0.0%
Ile	(I)	0	0.0%
Leu	(L)	0	0.0%
Lys	(K)	0	0.0%
Met	(M)	0	0.0%
Phe	(F)	0	0.0%
Pro	(P)	0	0.0%
Ser	(S)	0	0.0%
Thr	(T)	165	20.4%
Trp	(W)	0	0.0%
Tyr	(Y)	0	0.0%
Val	(V)	0	0.0%
Pyl	(O)	0	0.0%
Sec	(U)	0	0.0%
	(B)	0	0.0%
	(Z)	0	0.0%
	(X)	0	0.0%

Total number of negatively charged residues (Asp + Glu): 0
Total number of positively charged residues (Arg + Lys): 0

Atomic composition:

Carbon	C	2365
Hydrogen	H	3922
Nitrogen	N	810
Oxygen	O	976
Sulfur	S	240

Formula: $C_{2365}H_{3922}N_{810}O_{976}S_{240}$
Total number of atoms: 8313

Extinction coefficients:

This protein does not contain any Trp residues. Experience shows that this could result in more than 10% error in the computed extinction coefficient.

Extinction coefficients are in units of M^{-1} cm^{-1}, at 280 nm measured in water.

Ext. coefficient 15000
Abs 0.1% (=1 g/l) 0.224, assuming all pairs of Cys residues form cystines

Ext. coefficient 0
Abs 0.1% (=1 g/l) 0.000, assuming all Cys residues are reduced

Estimated half-life:

The N-terminal of the sequence considered is C (Cys).

The estimated half-life is: 1.2 hours (mammalian reticulocytes, in vitro).
>20 hours (yeast, in vivo).
>10 hours (Escherichia coli, in vivo).

Instability index:

The instability index (II) is computed to be 59.97
This classifies the protein as unstable.

Aliphatic index: 21.60

Grand average of hydropathicity (GRAVY): 0.873

C11

Number of amino acids: 805

Molecular weight: 66154.09

Theoretical pI: 5.07

Amino acid composition: CSV format

Ala	(A)	193	24.0%
Arg	(R)	0	0.0%
Asn	(N)	0	0.0%
Asp	(D)	0	0.0%
Cys	(C)	223	27.7%
Gln	(Q)	0	0.0%
Glu	(E)	0	0.0%
Gly	(G)	225	28.0%
His	(H)	0	0.0%
Ile	(I)	0	0.0%
Leu	(L)	0	0.0%
Lys	(K)	0	0.0%
Met	(M)	0	0.0%
Phe	(F)	0	0.0%
Pro	(P)	0	0.0%
Ser	(S)	0	0.0%
Thr	(T)	164	20.4%
Trp	(W)	0	0.0%
Tyr	(Y)	0	0.0%
Val	(V)	0	0.0%
Pyl	(O)	0	0.0%
Sec	(U)	0	0.0%
	(B)	0	0.0%
	(Z)	0	0.0%
	(X)	0	0.0%

Total number of negatively charged residues (Asp + Glu): 0
Total number of positively charged residues (Arg + Lys): 0

Atomic composition:

Carbon	C	2354
Hydrogen	H	3905
Nitrogen	N	805
Oxygen	O	970
Sulfur	S	223

Formula: $C_{2354}H_{3905}N_{805}O_{970}S_{223}$
Total number of atoms: 8257

Extinction coefficients:

This protein does not contain any Trp residues. Experience shows that this could result in more than 10% error in the computed extinction coefficient.

Extinction coefficients are in units of M^{-1} cm^{-1}, at 280 nm measured in water.

Ext. coefficient 13875
Abs 0.1% (=1 g/l) 0.210, assuming all pairs of Cys residues form cystines

Ext. coefficient 0
Abs 0.1% (=1 g/l) 0.000, assuming all Cys residues are reduced

Estimated half-life:

The N-terminal of the sequence considered is C (Cys).

The estimated half-life is: 1.2 hours (mammalian reticulocytes, in vitro).
>20 hours (yeast, in vivo).
>10 hours (Escherichia coli, in vivo).

Instability index:

The instability index (II) is computed to be 58.83
This classifies the protein as unstable.

Aliphatic index: 23.98

Grand average of hydropathicity (GRAVY): 0.870

D11

Number of amino acids: 808

Molecular weight: 66341.25

Theoretical pI: 5.07

Amino acid composition: CSV format

Ala (A)	191	23.6%
Arg (R)	0	0.0%
Asn (N)	0	0.0%
Asp (D)	0	0.0%
Cys (C)	223	27.6%
Gln (Q)	0	0.0%
Glu (E)	0	0.0%
Gly (G)	229	28.3%
His (H)	0	0.0%
Ile (I)	0	0.0%
Leu (L)	0	0.0%
Lys (K)	0	0.0%
Met (M)	0	0.0%
Phe (F)	0	0.0%
Pro (P)	0	0.0%
Ser (S)	0	0.0%
Thr (T)	165	20.4%
Trp (W)	0	0.0%
Tyr (Y)	0	0.0%
Val (V)	0	0.0%
Pyl (O)	0	0.0%
Sec (U)	0	0.0%
(B)	0	0.0%
(Z)	0	0.0%
(X)	0	0.0%

Total number of negatively charged residues (Asp + Glu): 0
Total number of positively charged residues (Arg + Lys): 0

Atomic composition:

Carbon	C	2360
Hydrogen	H	3914
Nitrogen	N	808
Oxygen	O	974
Sulfur	S	223

Formula: $C_{2360}H_{3914}N_{808}O_{974}S_{223}$
Total number of atoms: 8279

Extinction coefficients:

This protein does not contain any Trp residues. Experience shows that this could result in more than 10% error in the computed extinction coefficient.

Extinction coefficients are in units of M^{-1} cm^{-1}, at 280 nm measured in water.

Ext. coefficient 13875
Abs 0.1% (=1 g/l) 0.209, assuming all pairs of Cys residues form cystines

Ext. coefficient 0
Abs 0.1% (=1 g/l) 0.000, assuming all Cys residues are reduced

Estimated half-life:

The N-terminal of the sequence considered is C (Cys).

The estimated half-life is: 1.2 hours (mammalian reticulocytes, in vitro).
>20 hours (yeast, in vivo).
>10 hours (Escherichia coli, in vivo).

Instability index:

The instability index (II) is computed to be 58.05
This classifies the protein as unstable.

Aliphatic index: 23.64

Grand average of hydropathicity (GRAVY): 0.859

Reference

Gasteiger E., Hoogland C., Gattiker A., Duvaud S., Wilkins M.R., Appel R.D., Bairoch A.;
Protein Identification and Analysis Tools on the Expasy Server;
(In) John M. Walker (ed): The Proteomics Protocols Handbook, Humana Press (2005).
pp. 571-607
Full text - Copyright Humana Press.
